# Supplementary material for: Resolving the full spectrum of human genome variation using Linked-Reads
Source: Genome Res. 2019 Apr;29(4):635–45. doi: 10.1101/gr.234443.118 (PMC6442396; doi:10.1101/gr.234443.118)
Supplement: Supplemental Material [file supp_gr.234443.118_Supplemental_longranger-2.2.zip › longranger-2.2.tar.bz2!/lr2.2-public/lib/stan/cmdstan/stan/doc/api/html/check__consistent__size_8hpp_source.html]

Stan: src/stan/math/error\_handling/check\_consistent\_size.hpp Source File


|  |  |
| --- | --- |
|  | Stan  2.5.0  probability, sampling & optimization |


- Main Page
- Related Pages
- Namespaces
- Classes
- Files

- File List
- File Members

All Classes Namespaces Files Functions Variables Typedefs Enumerations Enumerator Friends Macros Pages

- src
- stan
- math
- error\_handling

check\_consistent\_size.hpp

Go to the documentation of this file.

1 #ifndef STAN\_\_MATH\_\_ERROR\_HANDLING\_\_CHECK\_CONSISTENT\_SIZE\_HPP

2 #define STAN\_\_MATH\_\_ERROR\_HANDLING\_\_CHECK\_CONSISTENT\_SIZE\_HPP

3

4 #include <sstream>

5 #include <stan/math/error\_handling/dom\_err.hpp>

6 #include <stan/meta/traits.hpp>

7

8 namespace stan {

9  namespace math {

10

11  // NOTE: this will not throw if nan is passed in.

12  template <typename T, typename T\_result>

13  inline bool check\_consistent\_size(size\_t max\_size,

14  const char\* function,

15  const T& x,

16  const char\* name,

17  T\_result\* result) {

18  size\_t x\_size = stan::size\_of(x);

19  if (is\_vector<T>::value && x\_size == max\_size)

20  return true;

21  if (!is\_vector<T>::value && x\_size == 1)

22  return true;

23

24  std::stringstream msg;

25  msg << " dimension=%1%, expecting dimension of either 1 or "

26  << "max\_size=" << max\_size

27  << "; a vectorized function was called with arguments of different "

28  << "scalar, array, vector, or matrix types, and they were not "

29  << "consistently sized; all arguments must be scalars or "

30  << "multidimensional values of the same shape.";

31

32  std::string tmp(msg.str());

33

34  return dom\_err(function,x\_size,name,

35  tmp.c\_str(), "",

36  result);

37  }

38

39  }

40 }

41 #endif

dom\_err.hpp

stan::math::dom\_err

bool dom\_err(const char \*function, const T &y, const char \*name, const char \*error\_msg, const T\_msg error\_msg2, T\_result \*result)

**Definition:** dom\_err.hpp:36

stan::is\_vector

**Definition:** traits.hpp:83

traits.hpp

stan::math::check\_consistent\_size

bool check\_consistent\_size(size\_t max\_size, const char \*function, const T &x, const char \*name, T\_result \*result)

**Definition:** check\_consistent\_size.hpp:13

stan::size\_of

size\_t size\_of(const T &x)

**Definition:** traits.hpp:186

stan::max\_size

size\_t max\_size(const T1 &x1, const T2 &x2)

**Definition:** traits.hpp:191


---

[ Stan Home Page ]

*© 2011–2014,
Stan Development Team.*
